# High-Throughput Analysis of Calcium Signalling Kinetics in Astrocytes Stimulated with Different Neurotransmitters

**DOI:** 10.1371/journal.pone.0026889

**Published:** 2011-10-25

**Authors:** Laura R. James, Simon Andrews, Simon Walker, Paula R. S. de Sousa, Aaron Ray, Noah A. Russell, Tomas C. Bellamy

**Affiliations:** 1 Laboratory of Molecular Signalling, The Babraham Institute, Babraham, Cambridge, United Kingdom; 2 Bioinformatics Group, The Babraham Institute, Babraham, Cambridge, United Kingdom; 3 School of Biomedical Sciences, University of Nottingham Medical School, Queen's Medical Centre, Nottingham, United Kingdom; 4 Schools of Biology and Electrical and Electronic Engineering, Institute of Biophysics, Imaging and Optical Science, University of Nottingham, Nottingham, United Kingdom; University of Cincinnati, United States of America

## Abstract

Astrocytes express a wide range of receptors for neurotransmitters and hormones that are coupled to increases in intracellular Ca^2+^ concentration, enabling them to detect activity in both neuronal and vascular networks. There is increasing evidence that astrocytes are able to discriminate between different Ca^2+^-linked stimuli, as the efficiency of some Ca^2+^ dependent processes – notably release of gliotransmitters – depends on the stimulus that initiates the Ca^2+^ signal. The spatiotemporal complexity of Ca^2+^ signals is substantial, and we here tested the hypothesis that variation in the kinetics of Ca^2+^ responses could offer a means of selectively engaging downstream targets, if agonists exhibited a “signature shape” in evoked Ca^2+^ response. To test this, astrocytes were exposed to three different receptor agonists (ATP, glutamate and histamine) and the resultant Ca^2+^ signals were analysed for systematic differences in kinetics that depended on the initiating stimulus. We found substantial heterogeneity between cells in the time course of Ca^2+^ responses, but the variation did not correlate with the type or concentration of the stimulus. Using a simple metric to quantify the extent of difference between populations, it was found that the variation between agonists was insufficient to allow signal discrimination. We conclude that the time course of global intracellular Ca^2+^ signals does not offer the cells a means for distinguishing between different neurotransmitters.

## Introduction

Astrocytes are non-excitable cells of the central nervous system, which nevertheless express a great many neurotransmitter and hormone receptors [1], [2]. These receptors commonly couple to Ca^2+^ signalling pathways, enabling astrocytes to detect transmitters released by the neuronal network and circulating hormones in the vasculature. Once triggered, increases in Ca^2+^ concentration activate downstream effectors such as transcription factors, kinases and exocytotic machinery [3]. Astrocytes are thus equipped to dynamically respond to signals in their microenvironment by changing cell morphology, gene expression profile, and releasing gliotransmitters to signal, in turn, to neurons, vascular cells and other glia. These signalling pathways regulate the many roles of astrocytes in the brain – metabolic support of neurons, buffering of K^+^ concentration, reactive gliosis in response to injury and maintenance of the blood brain barrier [4]–[7] – but they also raise the prospect that bidirectional communication between neurons and glia may play a computational role in the healthy brain [8], [9].

The form that a putative computational code in astrocyte Ca^2+^ signalling may take is the subject of much debate. One question raised by the number of receptors coupled to Ca^2+^ elevation in astrocytes is how selectivity of function can be maintained. The appropriate physiological response of a cell *in situ* to glutamate (a fast excitatory transmitter) may be expected to differ from its response to ATP (a diffusely acting inhibitory transmitter), or histamine (a neurotransmitter and inflammatory messenger) for example, and yet all of these agonists have been shown to trigger Ca^2+^ signals in glia [2].

One possibility is that the spatial range of Ca^2+^ signals differs with the receptor type that initiates it. Such spatially-restricted signalling is certainly known for Ca^2+^ microdomains that locally trigger exocytosis, but many transmitters are capable of triggering “global” Ca^2+^ signals that spread throughout the cell and even between cells via gap junctions. Another possibility that has been explored is that the interval between repetitive Ca^2+^ oscillations generated by a stimulus may encode information regarding agonist concentration [10]. This concept of frequency modulation has been investigated in many cell types, most notably hepatocytes [11], but such a coding mechanism would require persistent exposure to agonist, a scenario unlikely to be encountered for neurotransmitters under physiological conditions. A third possibility that has received less attention to date is that the temporal “shape” of a Ca^2+^ response could carry information about the stimulus that triggered it – that the kinetics of cytosolic Ca^2+^ concentration could vary depending on the nature of the initiating stimulus.

Such variation in kinetics is observed between different cell types, where the expression of different levels of the channels, pumps and buffers of the cellular Ca^2+^ signalling apparatus can generate Ca^2+^ oscillations of strikingly different duration and range [12]. However, there is also scope in principle – given the spatiotemporal complexity of Ca^2+^ dynamics – to encode information about the type of agonist and/or its concentration into the timing of a global Ca^2+^ response, even within a single cell type [13]. For example, variation between neurotransmitters in the balance of expression levels of ionotropic and metabotropic receptors, the strength of G-protein coupling to phospholipase C, the kinetics of receptor desensitization, and the possibility for parallel activation of additional signalling pathways that feedback to the Ca^2+^ signalling apparatus, could all generate diversity in the dynamics of cytosolic Ca^2+^ concentration. For example, in the case of glutamate, different temporal profiles of Ca^2+^ response may be generated by Ca^2+^ influx through GluA2-deficient AMPA receptors versus InsP_3_-dependent release of Ca^2+^ from internal stores after activation of mGluRs. Additionally, despite mGluR1, P2Y and H1 receptors all being coupled to G_q_ proteins, activation of the different receptors may generate changes in InsP_3_ concentration (and associated Ca^2+^ release) with differing kinetics, due to variation in desensitization kinetics, receptor density, or the strength of coupling to G_q_ proteins between the three receptor types. Co-expression of different receptor classes, such as G_q_-coupled H1 and G_s_-coupled H2 receptors may also generate different Ca^2+^ kinetics than expression of only a single receptor class, if crosstalk between the signalling cascades alters the activity of some of the many components of the Ca^2+^ signalling apparatus. Such mechanisms of signal diversification could allow astrocytes exposed to transient concentrations of neurotransmitters *in situ* to encode information about the nature of the stimulus into the kinetics of the provoked response.

In this study, we sought to test the concept that astroglial cells could discriminate between different agonists, at different concentrations, by systematic variation in the time course of the global Ca^2+^ signals that they generate. We examined the long-term response of the cells to tonic application of glutamate, ATP and histamine at various concentrations, but also used a high-throughput imaging technique to analyse the short-term Ca^2+^ response of naïve cells to the agonists. We compared astrocytes cultured from both cerebellum and cortex, to determine whether the dynamics of the Ca^2+^ signal elicited by the agonists varied in predictable ways. We found that although Ca^2+^ responses showed substantial heterogeneity in kinetics, the time course of the Ca^2+^ signal did not vary systematically between agonists, suggesting that the cells could not exploit this variability to discriminate between stimuli.

## Results

### Expression of ionotropic and metabotropic receptors in cultured astrocytes

**Figure 1:**
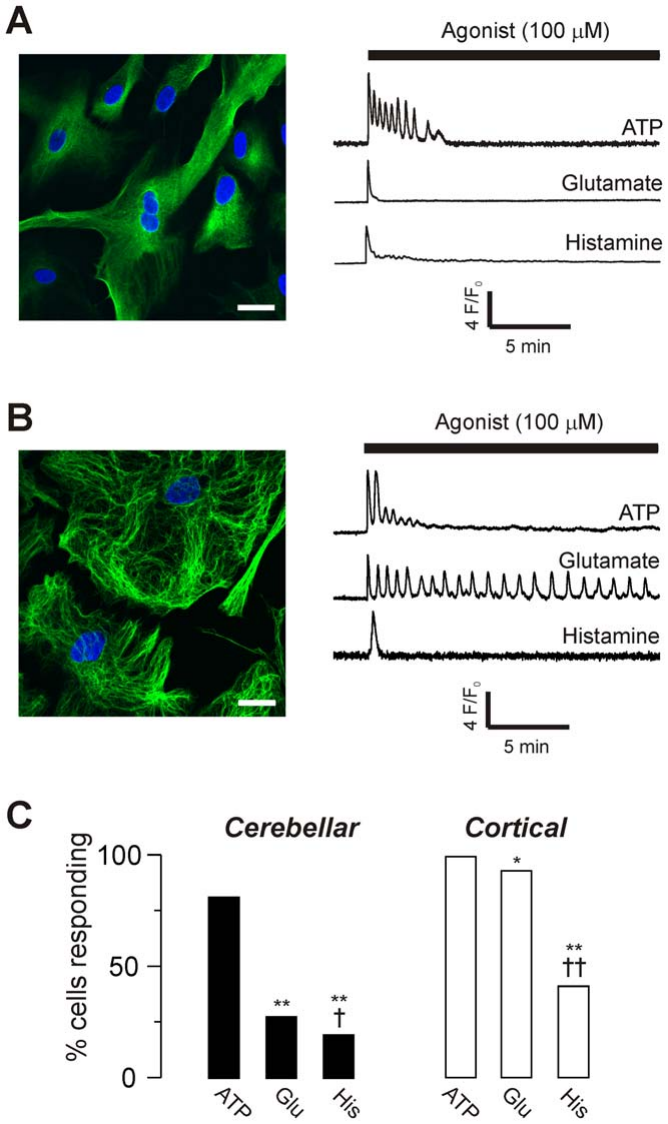
Ca^2+^ signals evoked by receptor agonists in cultured astrocytes. **A**) Left panel: Confocal image of cerebellar astrocytes stained for the astrocyte specific glial fibrillary acidic protein (GFAP, green) and a nuclear stain (DAPI, blue) captured using a point scanning confocal microscope. Scale bar = 20 µM. Right panel: Representative normalized fluorescence changes in single live cells loaded with fluo-4 AM and stimulated with 100 µM ATP, glutamate or histamine (as indicated). **B**) Confocal image and representative fluorescence changes in cortical astrocytes under the same conditions as in (A). **C**) Percentage of cells responding to each agonist (where response is defined as an increase in fluorescence of greater than 1.045 fold), for both cerebellar (left panel, filled bars) and cortical (right panel, unfilled bars) cultures. Data for each agonist are from 3–8 coverslips of cells, obtained from 2–3 culture preparations. Total cerebellar cells tested: 175 (100 µM ATP); 580 (100 µM glutamate), 161 (100 µM histamine). Total cortical cells tested: 129 (100 µM ATP); 504 (100 µM glutamate), 134 (100 µM histamine). Statistical significance of differences in the percentage of responding cells was tested by Fisher's exact test. *p <0.05 or ** p <0.001 compared to ATP, and †p <0.05 or ††p <0.001 compared to glutamate.

Primary cultures were prepared from cerebellum and cortex, and stained for the astrocyte marker glial fibrillary acidic protein (GFAP; Fig 1A,B). Greater than 95% of cells were GFAP positive. Live cells were loaded with the Ca^2+^ indicator fluo-4, and then exposed to ATP, glutamate and histamine for 20 min at a concentration of 100 µM. All three agonists evoked Ca^2+^ signals in both cerebellar and cortical astrocytes (Fig 1A–C).

**Figure 2:**
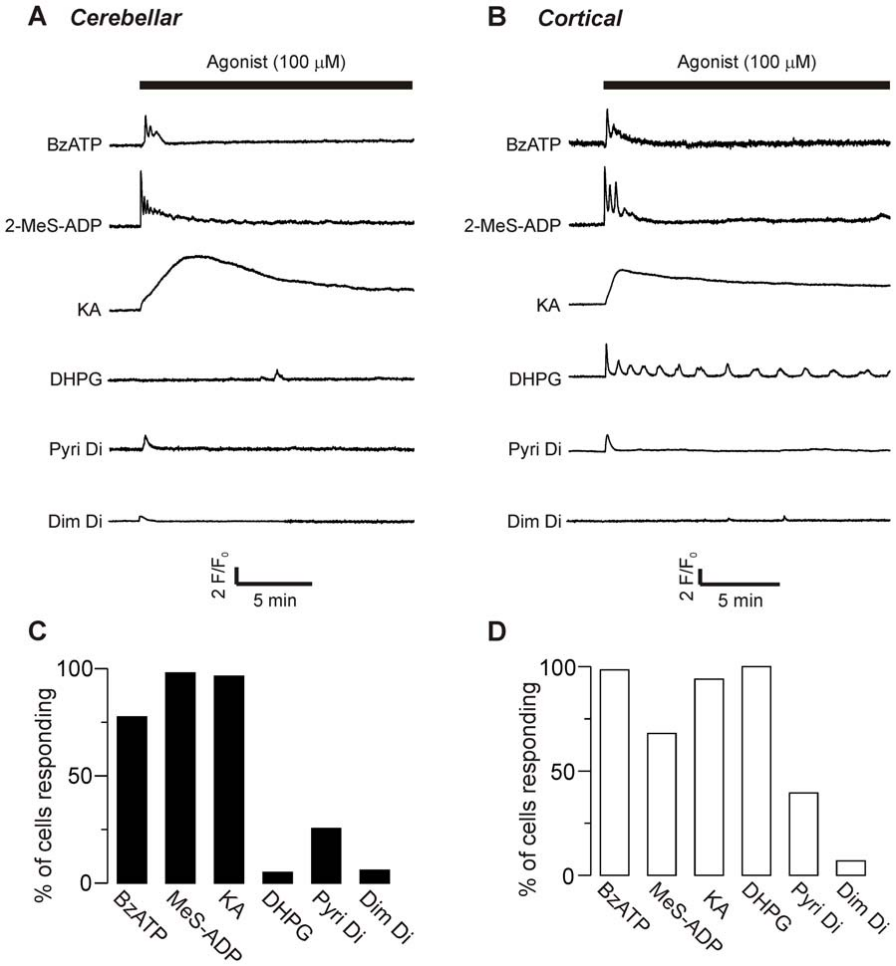
Pharmacological characterization of receptor types. Representative traces of changes in fluorescence intensity over time after addition of pharmacological agonists (at 100 µM) selective for P2X receptors (BzATP), P2Y receptors (2-MeS-ADP), AMPA/kainate receptors (KA), mGlu receptors (DHPG), H1 receptors (Pyri Di) and H2 receptors (Dim Di), in cerebellar (**A**) and cortical (**B**) cells. **C**) Percentage of cerebellar and **D**) cortical cells responding to each agonist. Data for each agonist are from 3–6 coverslips of cells, obtained from 2–3 culture preparations. Total cells tested for each agonist ranged from 64 to 202.

To identify the classes of receptors linked to Ca^2+^ elevation in the cells, we applied selective pharmacological agonists for ionotropic and metabotropic receptor subtypes, at a high concentration (100 µM; Fig 2). A significant percentage of cerebellar astrocytes responded (Fig 2A,C) to the iGluR agonist kainate (KA), the P2X agonist 2′(3′)-*O*-[4-Benzoylbenzoyl]adenosine-5′-triphosphate (BzATP), the P2Y agonist 2-[Methylthio]adenosine-5′-trihydrogen diphosphate (2-MeS-ADP), and the H1 agonist 2-pyridylethylamine dihydrochloride (Pyri di), but very few cells responded to the mGluR1 agonist (*S*)-3,5-Dihydroxyphenylglycine (DHPG), or the H2 agonist dimaprit dihydrochloride (Dim di). Cortical astrocytes gave similar responses (Fig 2B,D), except that nearly all cells also responded to the mGluR agonist DHPG.

Collectively, these results suggest that in cortical astrocytes glutamate and ATP provoke a mixed ionotropic and metabotropic response and histamine a metabotropic-only response; whereas cerebellar astrocytes will have a mixed response to ATP, an ionotropic-only response to glutamate, and a metabotropic-only response to histamine. This suite of possible routes for Ca^2+^ mobilization may mean that the cells have the potential to vary the dynamics of Ca^2+^ signalling evoked by different receptor agonists.

### Classification of responses to persistent stimulation

**Figure 3:**
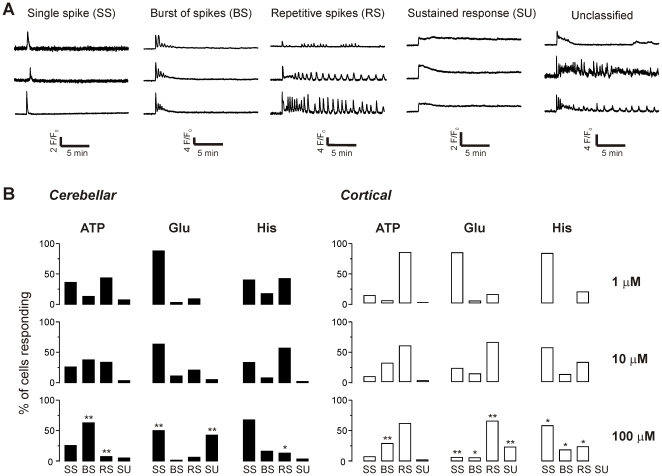
Classification of Ca^2+^ responses to long-term stimulation of astrocytes with agonists. **A**) Representative traces of Ca^2+^ responses in each class: single spikes (SS), a burst of spikes (BS), repetitive spikes (RS) or a sustained increase in Ca^2+^ (SU), as well as responses that did not fall into any category (see Methods). **B**) The percentage of responses falling into each category is illustrated. Data for each agonist at each concentration are from 3–8 coverslips of cells, obtained from 2–3 culture preparations. The total number of responding cells for cerebellar astrocytes (left panels) n = 122 (1 µM ATP); 307 (10 µM ATP); 142 (100 µM ATP); 33 (1 µM glutamate); 101 (10 µM glutamate); 160 (100 µM glutamate); 40 (1 µM histamine); 51 (10 µM histamine); 31 (100 µM histamine). For cortical astrocytes (right panels) n = 138 (1 µM ATP); 118 (10 µM ATP); 128 (100 µM ATP); 93 (1 µM glutamate); 168 (10 µM glutamate); 468 (100 µM glutamate); 11 (1 µM histamine); 50 (10 µM histamine); 55 (100 µM histamine). Statistical significance of differences between the proportion of cells in each class due to concentration of agonist was tested for each agonist by Fisher's exact test. *p <0.05 or ** p <0.001 for 100 µM agonist versus 1 µM.

As illustrated in Fig 1, when stimulating cells with 100 µM ATP, glutamate or histamine there was substantial variation evident in the time course of the Ca^2+^ response. Some cells gave only a single spike in response to persistent agonist exposure, others gave a burst of spikes that diminished in amplitude and lasted only a few minutes, others exhibited repetitive oscillations, and some showed persistent increases in Ca^2+^ concentration for the duration of the stimulus. To investigate whether the variation in response correlated with agonist type or concentration, we analysed the responses of both cerebellar and cortical astrocytes to ATP, glutamate and histamine at three different concentrations (1, 10 and 100 µM) and classified the responses into four broad categories (Fig 3A): a single spike (SS), a burst of spikes (BS), repetitive spikes (RS), or a sustained response (SU). A small percentage of cellular responses did not fall into these four categories (0.14% of total). These ambiguous cells (examples in Fig 3A) were excluded from further analysis.

For both cell types, all classes of response were observed for each of the three agonists, with the exception of histamine in cortical astrocytes which did not evoke sustained responses at any concentration (Fig 3B). There was a tendency for the percentage of cells in the different classes to vary as agonist concentration increased. To test for statistical significance, Fisher's exact test was used to compare the distribution of response classes at 1 µM and 100 µM for each of the agonists. In cerebellar astrocytes, there was a significant increase in burst spiking and a decrease in repetitive spiking with ATP, a decrease in single spiking and increase in sustained responses for glutamate, and a decrease in repetitive spiking for histamine (Fig 3B). For cortical astrocytes, there was an increase in burst spiking for ATP, a decrease in single spiking, but increase in burst spiking, repetitive spiking and sustained responses for glutamate, and a decrease in single spiking but increase in burst spiking and repetitive spiking for histamine (Fig 3B).

To compare the different agonists, Fisher's test was carried out for each class of response at 100 µM, to determine whether the likelihood of a particular class corresponding to a particular agonist was greater than chance (Table 1). For cerebellar astrocytes, the order of likelihood was as follows: for single spikes, ATP = His>Glu; for bursts, ATP>Glu>His; for repetitive spikes there were no significant differences; for sustained responses, Glu>ATP = His. For cortical astrocytes, the order was: for single spikes, His>ATP = Glu; for bursts, ATP>His = Glu; for repetitive spikes, ATP = Glu>His; for sustained responses, Glu>ATP = His.

**Table 1:** Tests of significant differences between classes of response evoked by different agonists.

Cerebellar astrocytes					Cortical astrocytes				
Condition	SS	BS	RS	SU	Condition	SS	BS	RS	SU
ATP vs Glu	**<0.0001**	**<0.0001**	0.82	**<0.0001**	ATP vs Glu	0.69	**<0.0001**	0.15	**<0.0001**
ATP vs His	**<0.0001**	**<0.0001**	0.28	1	ATP vs His	**0.0036**	**<0.0001**	**<0.0001**	0.079
Glu vs His	0.079	**0.0014**	0.25	**<0.0001**	Glu vs His	**<0.0001**	0.85	**<0.0001**	**<0.0001**

P values calculated using Fisher's exact test for each class of response in comparison of the agonists ATP, glutamate and histamine at 100 µM. SS = single spike, BS = burst of spikes, RS = repetitive spikes, SU = sustained response. Significant results (p<0.05) are in bold.

The lack of a clear trend in these results suggests that the pattern of spiking during persistent stimulation is unlikely to be the basis of a discriminatory mechanism. However, persistent exposure of astrocytes to neurotransmitter is a situation unlikely to occur *in vivo*, except under pathological conditions. We therefore examined the immediate response of naïve cells to agonist exposure, using a high-throughput technique.

### High throughput analysis of initial responses

**Figure 4:**
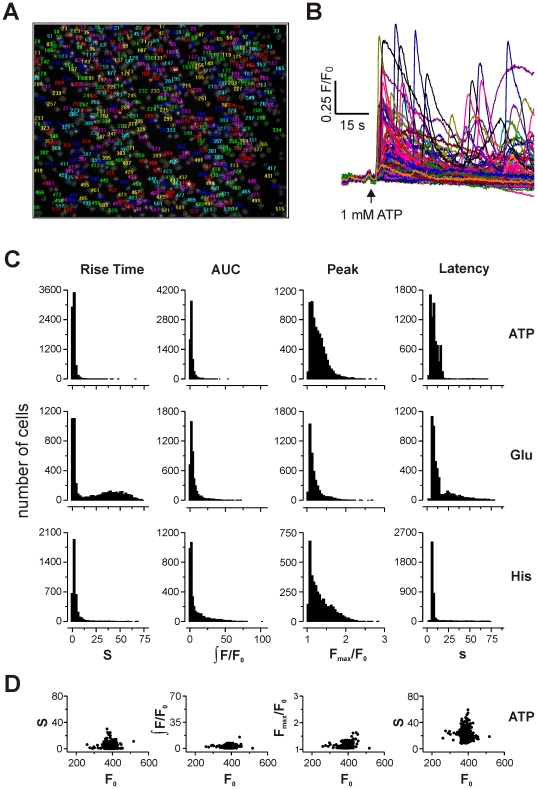
High throughput analysis of short-term Ca^2+^ responses. **A**) Example of a typical field of astrocytes labelled with Hoescht 33342 stain, after automated segmentation of regions of interested centred on cell nucleus. **B**) Changes in fluo-4 fluorescence intensity for all responding cells in a typical well stimulated with 1 mM ATP. **C**) Population histograms of the range of kinetic parameter values (rise time, area under curve, peak, and latency, as indicated above columns) observed in cerebellar astrocytes after stimulation with 1 mM ATP (top row), glutamate (middle row) or histamine (bottom row). Rise time and latency are measured in seconds (s), peak amplitude as maximal fold change in fluorescence intensity over baseline (F_max_/F_0_), and area under curve as the integral of F/F_0_ over time (see Methods for further details). **D**) Scatter plots of parameter value against initial fluorescence intensity (F_0_) for a typical well stimulated with 1 mM ATP.

Astrocytes from cerebellum or cortex were cultured on 96 well plates and loaded with the nuclear stain hoechst and the Ca^2+^ indicator fluo-4, to allow imaging after stimulation with glutamate, ATP and histamine over a range of concentrations, using a BD Pathway imaging system (Fig 4A,B). The initial response (100 s) after addition of agonist was recorded and analysed for four kinetic parameters using bespoke software: rise time (10% to 90% of maximum), peak (maximal fold change), area under curve (integrated fold change), and latency of response (time for addition to 10% of maximum). This method allowed us to accumulate data from >750,000 cells.

Plotting histograms for the distribution of Ca^2+^ responses reveals two key observations: that the heterogeneity of kinetic parameters is substantial, even within the same treatment condition, and that the range of responses does not have a normal distribution, instead having a clear rightward skew (Fig 4C). Accordingly, standard parametric tests for comparison of differences in mean values are inappropriate.

As a control for the influence of Ca^2+^ indicator buffering on Ca^2+^ kinetics, we plotted fluorescence intensity at rest (F_0_, as an index of indicator concentration) against each of the kinetic parameters examined (Fig 4D). There was no obvious correlation between F_0_ and rise time, area under curve, peak amplitude or latency, for any stimulus, suggesting that differential loading of cells was not a significant factor in the heterogeneity of resulting Ca^2+^ kinetics.

### Analysis of difference

To address the feasibility of cells discriminating between agonists on the basis of a given kinetic parameter, it seems insufficient to simply test for difference using a non-parametric test. If two populations of signals have largely overlapping but differently-shaped distributions, it is unlikely that a cell could discriminate between the signals in a single trial. Under these circumstances, a statistical test would assess the probability that the populations were different, but not *how* different, nor whether the difference was meaningful in terms of signal discrimination. To address this, we devised a simple metric to quantify the difference between two populations: population *A* was subtracted from population *B*, and the summed absolute difference was halved (see Methods). This gives a value for difference that ranges from 0 (identical populations) to 1 (no overlap).

**Figure 5:**
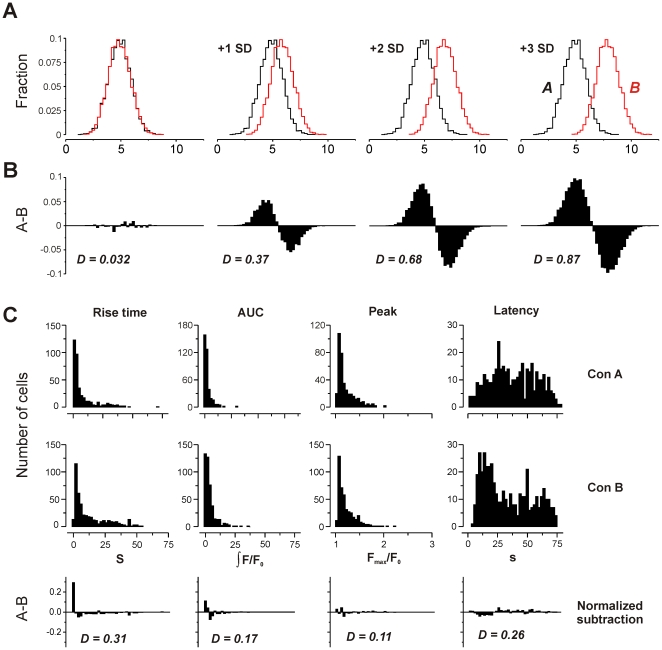
Measurement of difference between populations. **A**) Two populations of idealized data (Gaussian distribution, mean = 5, SD = 1, area = 1, n = 5000) were offset by multiples of the standard deviation. **B**) Subtraction of the (normalized) population *B* from population *A* shows increasing difference. Quantification of difference (see Methods) gives a value of *D* that rises as overlap between populations decreases. **C)** Histograms of kinetic parameters from typical control wells that received no stimulus (spontaneous Ca^2+^ signalling in cerebellar astrocytes receiving buffer exchange without agonist). A comparison was made of two control populations (Con A and Con B) that served as zero agonist controls in different imaging experiments. Note broad range of latencies, suggesting no correlation of spontaneous Ca^2+^ signals with mixing time. Lower panels show subtraction histograms and calculated *D* values for these spontaneous signals.

Carrying out such an analysis on simulated data (random data sets sampled from a Gaussian distribution) shows that progressively increasing the difference between population means by multiples of the standard deviation (SD) increases the value of *D* (Fig 5A,B). When the populations were offset by 1 SD, *D* = 0.37. Increasing the offset to 2 SD gives *D* = 0.68, and 3 SD gives *D* = 0.87. This gives a guide to interpretation, as values of *D* obtained from real data (Gaussian or non-Gaussian) can be compared to known overlap of the idealized data.

As a second guide for interpretation, we made a comparison of different wells of cells that were used as buffer controls for the imaging experiments. Each experiment had such a control to test for mixing artefacts and spontaneous activity. Some cells exposed to simple buffer exchange exhibited Ca^2+^ spikes, but they did not correlate with mixing time, suggesting they arose from spontaneous activity rather than mechanical artefacts (Fig 5C). We assumed that the intrinsic activity would have a common underlying mechanism, and so made a comparison of population responses for the buffer controls for each agonist. We took this to be a measure of random variation – that is a baseline of difference due to inherent biological noise or experimental variation rather than due to differing stimuli. *D* ranged from 0.03 to 0.31 (Table 2), with a mean of 0.15±0.10 SD, suggesting that any value ≤0.15 could be considered as background variation.

**Table 2:** Differences in kinetic parameters for spontaneous Ca^2+^ signals in unstimulated cells.

Cerebellar astrocytes					Cortical astrocytes				
Condition	Rise	AUC	Peak	Lat	Condition	Rise	AUC	Peak	Lat
Con 1 vs Con 2	0.31	0.17	0.11	0.26	Con 1 vs Con 2	0.092	0.048	0.043	0.11
Con 1 vs Con 3	0.30	0.28	0.27	0.28	Con 1 vs Con 3	0.085	0.057	0.033	0.10
Con 2 vs Con 3	0.19	0.17	0.24	0.28	Con 2 vs Con 3	0.078	0.057	0.040	0.12

Three control populations (cells exposed to buffer exchange in the absence of agonist) were compared. Values of difference (*D*) were calculated as outlined in Methods. Lower values indicate a greater degree of similarity between the distributions.

### Comparison of agonists, concentrations and brain regions

The initial hypothesis to be tested was that agonists differed in the kinetic profile of their Ca^2+^ response. At the highest concentration of ATP, glutamate and histamine tested (1 mM), the population responses were skewed and largely overlapping, regardless of stimulus (Fig 4C). Subtraction of populations give the difference metric *D*, with an overall mean of 0.23±0.12 SD (range 0.037 to 0.53; Table 3).

**Table 3:** Differences in kinetic parameters for Ca^2+^ signals evoked by different agonists.

Cerebellar astrocytes					Cortical astrocytes				
Condition	Rise	AUC	Peak	Lat	Condition	Rise	AUC	Peak	Lat
Glu vs ATP	0.45	0.28	0.26	0.32	Glu vs ATP	0.15	0.23	0.037	0.27
His vs ATP	0.23	0.27	0.19	0.53	His vs ATP	0.40	0.16	0.11	0.46
Glu vs His	0.46	0.24	0.28	0.41	Glu vs His	0.26	0.13	0.10	0.38

Three agonists: ATP, glutamate (glu) and histamine (his) were compared at a concentration of 1 mM. Values of difference (*D*) were calculated as outlined in Methods. Lower values indicate a greater degree of similarity between the distributions.

**Figure 6:**
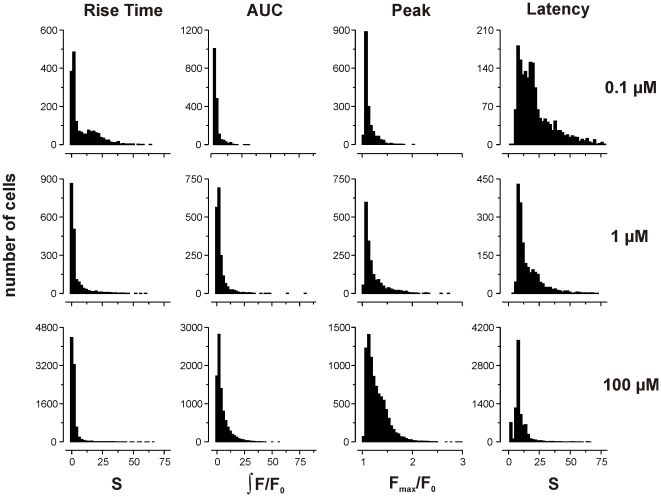
Effect of agonist concentration on kinetic parameters. Population histograms of the range of kinetic parameters observed in cerebellar astrocytes stimulated with 0.1 µM (top row), 1 µM (middle row) and 100 µM (bottom row) ATP. Rise time and latency are measured in seconds (s), peak amplitude as maximal fold change in fluorescence intensity over baseline (F_max_/F_0_), and area under curve as the integral of F/F_0_ over time (see Methods for further details). Difference was measured for all agonists. Results are summarized in Table 4.

A similar result was observed when comparing Ca^2+^ responses for each agonist (ATP, glutamate and histamine) at three different concentrations (10, 100 and 1000 µM). The population response did not appear to differ significantly with agonist concentration, beyond a tendency for longer latencies to be lost at higher concentrations (Fig 6). In keeping with this qualitative observation, *D* had overall mean of 0.22±0.13 SD (range 0.04 to 0.69; Table 4).

**Table 4:** Differences in kinetic parameters for Ca^2+^ signals evoked by different concentrations of agonist.

Cerebellar astrocytes					Cortical astrocytes				
**ATP (µM)**	**Rise**	**AUC**	**Peak**	**Lat**	**ATP (µM)**	**Rise**	**AUC**	**Peak**	**Lat**
10 vs 100	0.11	0.057	0.096	0.17	10 vs 100	0.16	0.12	0.22	0.19
10 vs 1000	0.22	0.24	0.12	0.34	10 vs 1000	0.14	0.19	0.32	0.43
100 vs 1000	0.12	0.24	0.04	0.35	100 vs 1000	0.14	0.093	0.14	0.47
**Glu (µM)**	**Rise**	**AUC**	**Peak**	**Lat**	**Glu (µM)**	**Rise**	**AUC**	**Peak**	**Lat**
10 vs 100	0.17	0.16	0.12	0.24	10 vs 100	0.10	0.15	0.17	0.21
10 vs 1000	0.34	0.22	0.14	0.22	10 vs 1000	0.12	0.29	0.37	0.54
100 vs 1000	0.19	0.12	0.051	0.16	100 vs 1000	0.13	0.14	0.21	0.39
**His (µM)**	**Rise**	**AUC**	**Peak**	**Lat**	**His (µM)**	**Rise**	**AUC**	**Peak**	**Lat**
10 vs 100	0.31	0.13	0.088	0.45	10 vs 100	0.13	0.28	0.30	0.17
10 vs 1000	0.46	0.097	0.17	0.69	10 vs 1000	0.22	0.25	0.37	0.24
100 vs 1000	0.17	0.18	0.24	0.62	100 vs 1000	0.13	0.14	0.076	0.32

Three agonists: ATP, glutamate (glu) and histamine (his) were compared at three concentrations (10, 100 and 1000 µM as indicated). Values of difference (*D*) were calculated as outlined in Methods. Lower values indicate a greater degree of similarity between the distributions.

**Figure 7:**
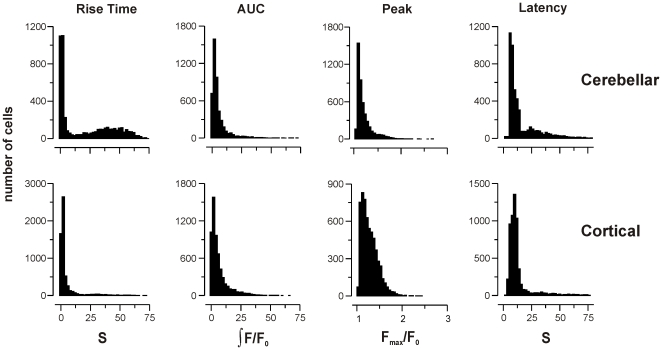
Effect of brain region on kinetic parameters. Population histograms of the range of kinetic parameters observed in cerebellar astrocytes (top row) and cortical astrocytes (bottom row) stimulated with 1 mM glutamate. Rise time and latency are measured in seconds (s), peak amplitude as maximal fold change in fluorescence intensity over baseline (F_max_/F_0_), and area under curve as the integral of F/F_0_ over time (see Methods for further details). Difference was measured for all agonists at 1 mM. Results are summarized in Table 5.

A final comparison was made between cerebellar and cortical astrocytes, stimulated with ATP, glutamate and histamine (Fig 7; Table 5). Again, there was no meaningful difference between the population responses, with mean *D* = 0.21±0.13 SD (range 0.03 to 0.48).

**Table 5:** Differences in kinetic parameters for Ca^2+^ responses evoked in cerebellar and cortical astrocytes.

Condition	Rise	AUC	Peak	Latency
Cerebellar vs cortical ATP	0.03	0.15	0.05	0.31
Cerebellar vs cortical Glu	0.35	0.11	0.28	0.25
Cerebellar vs cortical His	0.22	0.14	0.19	0.48

Cerebellar and cortical astrocytes stimulated with 1 mM ATP, glutamate (glu) and histamine (his) were compared. Values of difference (*D*) were calculated as outlined in Methods. Lower values indicate a greater degree of similarity between the distributions.

### Variation within cells

**Figure 8:**
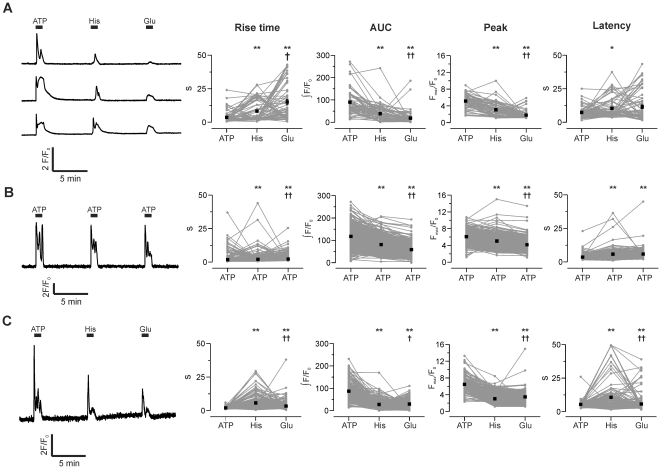
Variability of kinetic parameters within cells. **A**) Left panel: three representative traces of Ca^2+^ signals triggered by repetitive addition of ATP (100 µM), histamine (100 µM) and glutamate (100 µM) to the same population of cerebellar astrocytes. Right panels: kinetic parameters of responses in every cell (grey lines and symbols) and mean response ±s.e.m. (black symbols). **B**) A similar experiment to (A), but with three additions of ATP (100 µM). **C**) The same experiment as (A), in cortical astrocytes. Statistical significance of differences between parameter values were measured by Wilcoxon-signed-rank test. *p <0.05 or ** p <0.001 compared to first addition. †p <0.05 or ††p <0.001 compared to second addition.

The foregoing comparisons have been from data gathered from a large population of cells, with the assumption being that variation from cell to cell in Ca^2+^ kinetics would be less than the variation between agonists across the whole population (if the hypothesis was correct). It is possible, however, that the converse is true – that variation in kinetics between agonists may occur in each cell, but the differences between cells overwhelms the trend. This possibility is harder to test, as it requires repetitive addition of agonists to the same cell population; consequently, the cells will not be naïve for the later additions (and so store emptying, receptor desensitization, etc. may confound interpretation), and the protocol is less amenable to high-throughput screening. Nevertheless, we exposed astrocytes to 100 µM ATP, histamine and glutamate for 1 min sequentially with 7 min recovery period between additions, and then analyzed the response kinetics of those cells that responded to all three stimuli (Fig 8).

As expected from the high throughput experiments, variation in kinetic parameters for each agonist from cell to cell was large (Fig 8A). We used a Wilcoxon-signed-rank test to compare the kinetic parameters for each of the agonists. Mean rise time differed significantly in the order: ATP<histamine<glutamate, whereas peak and area under curve showed the opposite trend: ATP>histamine>glutamate (Fig 8A). To attempt to disentangle agonist-specific effects from the complications of repetitive stimulation, we repeated the experiment with three additions of ATP (Fig 8B). In this case, rise time increased only marginally for later additions, but peak and AUC showed a time-dependent decline. All changes were statistically significant, suggesting that the changes in kinetic parameters due to agonist specific effects will be difficult to separate from differences due to repeated stimulation. The same experiment was carried out in cortical astrocytes (Fig 8C). As with cerebellar astrocytes, kinetic parameters differed significantly, but this result may be due to repetitive addition rather than agonist-specific effects. These results highlight the problem of judging whether statistically significant differences in mean values are biologically significant differences in terms of signal discrimination.

## Discussion

### Spatiotemporal complexity of Ca^2+^ responses

It is twenty seven years since neurotransmitter receptors were first described in astrocytes [14], [15], and since that time, the number of Ca^2+^-linked receptors found to be expressed by the cells has expanded enormously. This abundance of physiological stimuli linked to Ca^2+^ elevation raises questions about how specificity of action can be maintained – it seems doubtful that the appropriate response of an astrocyte to a circulating hormone such as endothelin, should be the same as its response to a fast excitatory neurotransmitter such as glutamate, for example. In keeping with this argument, evidence is accumulating that downstream targets of Ca^2+^ can be selectively engaged, depending on the initial stimulus. The best current example is the release of “gliotransmitters” from astrocytes, a Ca^2+^ dependent process that is pronounced when cells are stimulated by PAR agonists, but more limited when cells are stimulated with P2Y receptor agonists, despite comparable Ca^2+^ responses [16]. The basis of this selectivity is not understood, but may explain the divergent results regarding the role of gliotransmission *in vivo* if the stimulus initiating Ca^2+^ elevation determines efficacy of release [17]. Similar selectivity has been found with astrocyte dependent neurovascular coupling – Ca^2+^-mobilizing stimuli can trigger either dilatation or constriction of the blood vessels that the astrocytes abut [18]-[20]. A recent example of how precise such selectivity can be, is the demonstration in HSG cells that activation of distinct transcription factors (NFAT and NF-κ-B) occurs depending on the composition of Ca^2+^ entry complexes during store-operated Ca^2+^ entry [21].

In this study we tested the hypothesis that the spatiotemporal complexity of Ca^2+^ signals offered a means for discrimination on the basis of the timing of the Ca^2+^ response. Our reasoning was that agonists may present a characteristic “signature” Ca^2+^ spike shape, which could offer a means for selectively engaging downstream targets. It has long been known that many cell types (including astrocytes) can exhibit kinetically complex Ca^2+^ signals, such as repetitive oscillations in Ca^2+^ concentration from basal levels, sinusoidal oscillations on a raised baseline, short bursts of oscillations, or prolonged high amplitude plateaux of Ca^2+^ [10]. This heterogeneity in Ca^2+^ dynamics can arise from several sources, but a major cause is the inherent stochasticity of the mechanisms underlying global Ca^2+^ signal generation.

Global Ca^2+^ signals arise from the amplification of elementary release events generated by the opening of a small number of clustered ER Ca^2+^ channels. It is the best known example of how the random behaviour of individual channel openings can be amplified to a whole cell scale, most notably in the variation in interspike intervals during baseline oscillations [22], [23]. Because this mechanism depends on amplification of stochastic processes, even relatively small perturbations in the expression levels of Ca^2+^ release channels, buffers and clearance pumps can give rise to dramatic changes in Ca^2+^ dynamics. This variability in the balance of release and extrusion mechanisms has been shown to result in intercellular heterogeneity of response to agonists in genetically identical cell populations [24]. Modelling studies have borne out the contention that simply changing the relative levels of expression of Ca^2+^ handling proteins is sufficient to account for large scale changes in global signal dynamics [23], [25].

In the case of astrocytes, however, there are additional sources of complexity. Gap-junctional coupling between cells can provide a route for diffusion of InsP_3_ and Ca^2+^ between adjacent cells. A study by Venance et al. [26] showed that inhibition of gap junctions decreased the heterogeneity of responses to α-adrenergic and muscarinic receptor agonists in striatal astrocytes. Similarly, the Ca^2+^-dependent release of gliotransmitters such as ATP can initiate paracrine (or autocrine) signalling in cultured astrocytes [27]. Accordingly, these mechanisms can give rise to secondary signalling events, wherein some cells within the population that do not respond to the initial signal are stimulated instead by neighbouring cells.

In principle, different neurotransmitters and hormones could engage these various sources of kinetic heterogeneity to varying degrees, and so bias the Ca^2+^ signal towards certain kinetic profiles. However, the evidence accumulated in this study suggests that the substantial heterogeneity in the kinetics of Ca^2+^ responses does not correlate with the initiating stimulus during either short- or long-term stimulation.

### Heterogeneity in astrocyte responses

During persistent exposure, there was a tendency for some agonists to favour a particular class of response (single spikes, bursts of spikes, repetitive oscillations or sustained increase in Ca^2+^); for example, glutamate in cerebellar astrocytes showed single spikes or sustained responses whereas ATP showed bursts of spikes or repetitive spikes. However, these trends were not robust; all classes of response were observed to differing degrees for all transmitters. No class could be taken to be characteristic (or predictive) of stimulation with a particular agonist. A similar lack of distinctiveness was observed when the initial response of cells was analysed – heterogeneity existed in the range of parameter values, but did not vary in a systematic manner with the type or concentration of stimulus (Fig 5). The variability between cells appeared to be greater than the variability within a given cell stimulated by different agonists (Fig 8), but no clear patterns emerged, suggesting that biological “noise” in expression levels of the Ca^2+^ signalling apparatus overwhelms any difference in the manner of coupling different agonist receptors to the global signalling cascade.

The hypothesis that Ca^2+^ signal shape allows discrimination between stimuli is therefore almost certainly incorrect. Assessing this hypothesis in a quantitative way has, however, raised some issues regarding analysis of discrimination in biochemical signalling pathways.

### Measuring signal difference

One ramification of the population responses we observed is that the kinetic parameters do not have a Gaussian distribution, which means that the use of parametric tests for assessing changes in mean parameter values for small sample sizes – a not uncommon practice – is inappropriate, and may give rise to spurious conclusions. Use of non-parametric tests may protect against this problem, but to answer the specific question raised by our hypothesis of *how* different two populations are (rather than a question of whether it is improbable that any apparent difference may have emerged by chance) a measure of the difference between the populations was needed.

The Kullback-Leibler divergence (*D*_KL_) is a metric derived from information theory and used in communications applications to assess signal degradation between a reference signal and the measured output signal. If signals are identical, the *D*_KL_ is zero, but as the signals diverge, *D*_KL_ increases, in principle to infinity. Application of this measure to biological data has been carried out [28], but a limitation of the metric is that it depends on the ratio of values between the two populations. For continuous data fitted with a known function, this is straightforward, but for discontinuous data (as in this study) any bin that has the value of zero in either population gives rise to an error. Rather than fitting an arbitrary function to our data to facilitate Kullback-Leibler analysis, we instead opted to use a simple subtraction metric (see Methods), that ranged from 0 (identical) to 1 (no overlap). This method confirmed the qualitative assessment that population distributions were overlapping with similar shape, but also allowed a quantitative measure of difference that could be compared to ideal and control data. For the majority of cases, the values of *D* were small (mean ≈0.2) and indistinguishable from control differences (mean = 0.15±0.1 SD). Although some comparisons returned higher values of *D* (maximum 0.69), this would be predicted due to chance, and there was no obvious pattern to suggest that a given kinetic parameter consistently showed large differences between agonists or concentrations (see Tables 2, 3, 4, 5). From the perspective of a cell *in vivo*, the transmitter type (or concentration) would be need to be reliably identified from the Ca^2+^ kinetics resulting from a single exposure to the agonist. This would require response distributions with negligible overlap and *D* values that approach 1. In an idealised example there is still considerable overlap at *D* values as high as 0.87 (Figure 5). None of the *D* values in this study were sufficiently high to support the idea that reliable discrimination between transmitters can be made on the basis of the temporal structure of the Ca^2+^ response.

### Physiological implications

The various sources of heterogeneity in astrocyte Ca^2+^ signalling in cultured cells are also known to be present *in vivo*. Consequently, it is likely that a similar diversity of dynamic responses would be present under physiological conditions, and that discrimination between different classes of neurotransmitters and hormones on kinetic grounds is unlikely to be feasible. This suggests that other mechanisms are in place. Spatially restricted “microdomain” signalling has been observed [29], [30], but does not address the question of how different stimuli that all evoke global signals can be differentiated. Coincident activation of other parallel signalling pathways is one possibility, given the scope for crosstalk between Ca^2+^ and other second messenger systems. Alternatively, it may be the case that such global signals merely convey to the cell that any of a range of external stimuli has reached a sufficient strength to cross a threshold, and so acts as a simple binary signal that cannot discriminate between different stimuli. This leaves unresolved the question of how astrocytes can mount selective responses to activation of different Ca^2+^ mobilizing receptors, but the timing of the Ca^2+^ response appears to be of little relevance.

## Materials and Methods

### Ethics statement

All experiments were performed in accordance with the guidelines set out in the code of practice for humane killing under Schedule 1 of the UK Home Office Animals (Scientific Procedures) Act 1986, and were approved by the Babraham Research Campus Animal Welfare, Experimentation, and Ethics Committee (Certificate of Designation PCD 80/4804).

### Preparation of cerebellar and cortical astrocyte cultures

Neonatal (P2) rat pups were sacrificed by cervical dislocation and decapitation. Whole brains were removed under sterile conditions. Using a dissection microscope, the cerebellum and cerebral cortices were removed and transferred to separate vessels containing ice cold dissection buffer composed of: Ca^2+^-and Mg^2+^-free Hank's HEPES-buffered saline solution (Invitrogen, Paisley, UK), 22 mM glucose, 20 mM HEPES, 100 units.ml^−1^ penicillin, 100 µg.ml^−1^ streptomycin, 0.1 mM ­L­-serine, 0.5 mM L-glutamine and 1 mM sodium pyruvate (pH adjusted to 7.2 with NaOH). Meninges and blood vessels were carefully removed using fine forceps. The dissected tissue was finely chopped and dissociated enzymatically for 45 minutes at 37°C in dissection buffer supplemented with 15 µg/ml papain. Cerebella and cortical tissue was subsequently mechanically dissociated using repeated trituration through fire-polished Pasteur pipettes in the presence of 20 mg/ml DNase in medium containing DMEM (Invitrogen, Paisley, UK), 4500 mg.l^−1^ glucose, 4 mM L-glutamine, 110 mg L-pyruvate, 10% foetal bovine serum, 50 µg/ml ­L-proline, 100 units.ml^−1^ penicillin, and 100 µg/ml streptomycin. Cells were plated at a density of 5×10^4^ cells/ml (cerebellar astrocytes) or 8×10^4^ cells/ml (cortical astrocytes) on poly-l-lysine coated coverslips for long-term stimulation experiments or 96 well imaging plates (Grenier, Stonehouse, UK) for high throughput experiments. Medium was changed 24 hours after plating, and every 3–4 days thereafter.

### Immunofluorescence and confocal imaging

Cerebellar and cortical astrocytes were fixed in PBS containing 2% paraformaldehyde and 0.05% glutaraldehyde for 20 minutes at room temperature. Cells were washed several times with phosphate buffered saline (PBS) before being permeabilized in PBS containing 0.2% triton X-100 for 15 minutes. Blocking of non-specific binding sites was achieved by incubation in PBS containing 0.1% triton-X-100, 10% normal goat serum and 1% bovine serum albumin. Cells were incubated with primary antibody (Rabbit anti-GFAP; Synaptic Systems, Göttingen, Germany) in this buffer for 1 hour at room temperature. After further washing, cells were incubated with secondary antibody (goat anti-rabbit IgG) conjugated to Alexa Fluor 488 indicator (Invitrogen, Paisley, UK) for a further hour. After a final wash, coverslips were mounted onto slides with Vectashield containing the DNA counterstain 4′-6′ diamidion-2-phenylindole (DAPI, Vector laboratories, Peterborough, UK).

Cells were imaged on a point-scanning confocal microscope (Zeiss LSM 510 META) with 1.40 na, 63×oil objective. Indicators were excited at 488 nm (Alexa Fluor 488) and 405 nm (DAPI) and emission detected in bandwidths of 505–550 nm (Alexa Fluor 488) 420–480 nm (DAPI).

### Live cell imaging of Ca^2+^ signals

For long-term stimulation experiments, astrocytes were loaded with 1 µM of the Ca^2+^ indicator fluo-4 AM (Invitrogen, Paisley, UK) for 30 min in buffer containing: 135 mM NaCl, 3 mM KCl, 10 mM HEPES, 15 mM D-glucose, 2 mM MgSO_4_ and 2 mM CaCl_2_. Cells were then left to de-esterify accumulated fluo-4 AM for a further 30 min before transferring to the stage of an Olympus IX70 inverted microscope (20×, 0.75 NA objective). Loaded cells were excited at 450/20 nm with an exposure time of 100 ms, imaged at a frame rate of 2 Hz, and fluorescence emission (535/50 nm) was detected with an ORCA-ER camera (Hamamatsu, Welwyn Garden City, UK). Camera and shutter (Cairn Research Limited, Faversham, UK) were controlled by Wasabi software (Hamamatsu, Welwyn Garden City, UK). Agonists were added by manual displacement of bath solution with excess buffer containing agonist at the indicated concentration.

For high throughput imaging, a BD Pathway 855 High-Content Cell Analyser (BD, Oxford, UK) was used (10×, 0.3 NA objective). For these experiments, cells were co-loaded with hoechst 33342 and fluo-4 AM. Fluo-4 fluorescence (excitation 470/50 nm, emission 515-530 nm) was imaged continuously at a frame rate of 8 Hz, and a single image of Hoescht staining (excitation at 380/20 nm, emission at 460–480 nm) was acquired at the end of the sequence. Solution exchange was automated, with a 1∶10 dilution of stock solution into each well with an addition time of 1 s, followed by a re-dispensing mixing step (at 20 µl/s), giving a total mixing time of 2 to 3 s.

### Image analysis

Regions of interest centred on the cell nucleus were defined manually for long-term stimulation experiments, or by segmentation based on the Hoescht image (using AttoVision software) for high-throughput experiments. Fluorescence intensity was expressed as the ratio of fluorescence at time *t* divided by mean intensity for 0–20 s before addition of agonist (F/F_0_).

Kinetic parameters were measured with bespoke software (Auger; Bioinformatics group, Babraham Institute). Cells were defined as responders (i.e. cells that demonstrate a global Ca^2+^ signal that reaches the nucleus) if F/F_0_ increased >1.045 fold (at least 3 SD above the pre-stimulation mean). **Peak amplitude** is the largest fold-change in fluorescence, **rise time** is the time taken to increase from 10% to 90% of peak, **latency** is the time taken from addition of agonist to 10% of peak, and **area under curve (AUC)** is the integral of fluorescence intensity from 10% of peak to the end of the experiment. Cells were excluded from further analysis if Ca^2+^ signals occurred before addition of agonist.

For spike counting, traces were smoothed by averaging 50 adjacent time points (6.25 s) and subtracting the result from the original time course. The spike threshold was set at 0.4, with a minimum width of 5 s. Mean interspike interval (ISI) was also determined. For classification of responses to long-term stimulation, the following criteria were used: **single spike** (no. of spikes = 1; AUC <500), **burst of spikes** (no. of spikes >1; ISI× no. of spikes ≤300; AUC >1000), **repetitive spikes** (no. of spikes >1; ISI×no. of spikes>300), and **sustained response** (no. of spikes = 1; AUC>500). This protocol successfully sorted 2216 cells into the four classes from a total population of 2247 (31 cells were not classified = 0.14%).

### Measurement of difference between histograms

To assess how different two populations are, we used a simple subtraction method to quantify the difference (*D*) between two normalized histograms *A* and *B*, with *n* bins:
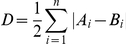



This yields a value from 0 (identical populations) to 1 (non-overlapping populations).

To provide a comparative scale showing how *D* varies with the extent of overlap, we used idealized surrogate data sets. Two normalized random Gaussian distributions (standard deviation = 1) were generated using Origin (Version 7.5; OriginLab, Northampton, USA). The difference between population means was then systematically increased, and *D* was calculated for each difference (see Figure 5). As the overlap between populations decreased, *D* increased, as expected.

### Statistical analysis

Differences in percentage of cells responding in a given class were measured with Fisher's exact test. Comparison of kinetic parameters in cells repetitively exposed to agonists was carried out with Wilcoxon signed-rank test. All statistical tests were performed with Prism (GraphPad, La Jolla, USA).
